# H558R, a common SCN5A polymorphism, modifies the clinical phenotype of Brugada syndrome by modulating DNA methylation of *SCN5A* promoters

**DOI:** 10.1186/s12929-017-0397-x

**Published:** 2017-12-04

**Authors:** Hiroya Matsumura, Yukiko Nakano, Hidenori Ochi, Yuko Onohara, Akinori Sairaku, Takehito Tokuyama, Shunsuke Tomomori, Chikaaki Motoda, Michitaka Amioka, Naoya Hironobe, Masaaki Toshishige, Shinya Takahashi, Katsuhiko Imai, Taijiro Sueda, Kazuaki Chayama, Yasuki Kihara

**Affiliations:** 1 Department of Cardiovascular Medicine, Hiroshima University Graduate School of Biomedical and Health Sciences, Hiroshima, Japan; 2 Laboratory for Digestive Diseases, Center for Genomic Medicine, RIKEN, Hiroshima, Japan; 3 Department of Gastroenterology and Metabolism, Division of Frontier Medical Science, Programs for Biomedical Research Graduate School of Biomedical Science, Hiroshima University, Hiroshima, Japan; 4 Department of Cardiovascular Surgery, Hiroshima University Hospital, Hiroshima, Japan

**Keywords:** Brugada syndrome, Single nucleotide polymorphism, *SCN5A*, Ventricular fibrillation

## Abstract

**Background:**

A common SCN5A polymorphism H558R (c.1673 A > G, *rs1805124*) improves sodium channel activity in mutated channels and known to be a genetic modifier of Brugada syndrome patients (BrS). We investigated clinical manifestations and underlying mechanisms of H558R in BrS.

**Methods and results:**

We genotyped H558R in 100 BrS (mean age 45 ± 14 years; 91 men) and 1875 controls (mean age 54 ± 18 years; 1546 men). We compared clinical parameters in BrS with and without H558R (H558R+ vs. H558R- group, *N* = 9 vs. 91). We also obtained right atrial sections from 30 patients during aortic aneurysm operations and compared *SCN5A* expression and methylation with or without H558R. H558R was less frequent in BrS than controls (9.0% vs. 19.2%, *P* = 0.028). The VF occurrence ratio was significantly lower (0% vs. 29.7%, *P* = 0.03) and spontaneous type 1 ECG was less observed in H558R+ than H558R- group (33.3% vs. 74.7%, *P* = 0.01). The *SCN5A* expression level was significantly higher and the methylation rate was significantly lower in sections with H558R (*N* = 10) than those without (0.98 ± 0.14 vs. 0.83 ± 0.19, *P* = 0.04; 0.7 ± 0.2% vs. 1.6 ± 0.1%, *P* = 0.004, respectively). In BrS with heterozygous H558R, the A allele mRNA expression was 1.38 fold higher than G allele expression.

**Conclusion:**

The SCN5A polymorphism H558R may be a modifier that protects against VF occurrence in BrS. The H558R decreased the SCN5A promoter methylation and increased the expression level in cardiac tissue. An allelic expression imbalance in BrS with a heterozygous H558R may also contribute to the protective effects in heterozygous mutations.

**Electronic supplementary material:**

The online version of this article (doi: 10.1186/s12929-017-0397-x) contains supplementary material, which is available to authorized users.

## Background

Brugada syndrome (BrS) is one of the main causes of sudden cardiac death in younger age groups resulting from ventricular fibrillation (VF) or polymorphic ventricular tachycardia [1]. A major causative gene of BrS is α-subunit of the cardiac sodium channel (Na_v_ 1.5) encoded by the *SCN5A* gene, which is located on chromosome 3p21 and consists of 101,617 bases and 28 exons encoding 2016 amino acids of Na_v_ 1.5 [2]. BrS is an autosomal dominant disease with incomplete penetrance and little is known about the mechanisms underlying the variability of penetrance. Past reports [3, 4] have suggested that a single nucleotide polymorphism (SNP) coexisting with a mutation might coordinate the penetrance. The common *SCN5A* polymorphism H558R (c.1673, A > G, *rs1805124*) has been reported to be such a genetic modulator; basic studies have shown that H558R improved sodium channel activity in mutated channels by repairing abnormal channel gating kinetics and membrane trafficking [3–6]. Clinically, they have reported that the presence of the minor allele A (H558R) improves ECG characteristics and the clinical phenotype among the carriers of a *SCN5A* mutation [7].

However, the mechanisms by which H558R rescues the *SCN5A* mutations are controversial and remain to be defined. In this study, we investigated the effects of H558R on the clinical manifestations and underlying mechanisms in BrS.

## Methods

### Participants

The study population comprised consecutive 100 patients (mean age at diagnosis of 45 ± 14 years; 91 men) diagnosed with BrS between May 1995 and November 2014 and 1875 normal controls (mean age 54 ± 18 years; 1546 men). All participants were Japanese and unrelated.

BrS was definitively diagnosed based on the 2013 HRS/EHRA/APHRS consensus statement [8]. Right atrial sections were obtained for expression analysis from 30 patients (17 men and 13 women; mean age 67 ± 12 years) during aortic aneurysm operations at our hospital. The Institutional Ethics Committee of the Graduate School of Biomedical Science at Hiroshima University approved all procedures, including the use of human tissue. Written informed consent was obtained from all participants.

### Genotyping of *rs1805124* in BrS patients and normal controls

Peripheral blood was obtained from all the subjects. Genomic DNA was extracted from leukocytes using a QIAamp DNA Blood Mini Kit (QIAGEN, Hilden, Germany) following the normal protocol. We genotyped *rs1805124* in all 100 BrS patients and 1875 healthy control subjects using the TaqMan assay, as described previously, [9, 10] and compared the frequency of H558R occurrence between the BrS patients and controls. We also genotyped *rs1805124* in the 30 patients who underwent aortic aneurysm operations for the expression study. For typing H558R (*rs1805124*), we used forward primer: TTTGGACTTGGCACTGGTGAT and reverse primer: AGACCTGGGTTCTGAAGCAGATT. We used the Invader oligo: GGGGGAGAGCGAGAGCCACCT, signal prove-G: CGCGCCGAGGACACATCACTGCTGGTG, and signal prove-T: ATGACGTGGCAGACGCACATCACTGCTGGT.

### Sequence analysis of *SCN5A* in BrS patients

Using Go Taq (Promega, Madison, WI, USA), all *SCN5A* coding regions were amplified by polymerase chain reaction (PCR) from 2.5 ng genomic DNA using our original primers. These amplified coding regions were directly resequenced from both directions using an ABI PRISM 310 Genetic Analyzer (Applied Biosystems, Foster City, CA, USA) to identify mutations and polymorphisms. Information about the *SCN5A* primers is presented in Additional file 1: Table S1.

### Analysis of gene expression and DNA Methylation of promoters in the *SCN5A* gene

The total RNA and genome DNA were isolated from the right atrial sections using an All Prep DNA/RNA/miRNA universal kit (QIAGEN). Quantitative reverse transcription PCR of the *SCN5A* mRNA was performed by QX200 Droplet Digital PCR (dd-PCR) system (Bio-Rad, Hercules, CA, USA) as described previously, using *GAPDH* as a reference gene [11, 12].

The primer sequences used for *SCN5A* were 5′**-** TTGCAGATGATGAAAACAGCACAG -3′ and 5′-GGCCAGGGCACCAGCA**-**3′, and for *GAPDH* were 5′**-**GTCTCCTCTGACTTCAACAGCG-3′ and 5′**-**ACCACCCTGTTGCTGTAGCCAA**-**3′.

The H558R was detected with the FAM probe: 5′-TGATGTGTGGTGGCT-3′ and the VIC probe: 5′-ATGTGCGGTGGCT-3′. The dd-PCR data were analyzed with Quanta Soft analysis software (Bio-Rad), and the quantification of either the G allele or A allele of cDNA and genome DNA in patients with heterozygous H558R was presented as the number of copies per microliter of PCR mixture.

We also examined the epigenetics of *SCN5A* in 16 CpG dinucleotides in the *SCN5A* promoter (capital means methylation site): tgtgtgtgtg tgtatactct ggCgggtgct ggtgtgtatg ccagtgtttg ttaatgtgag cctgtcCgCg tcCgCgtggg tggccatctg tggtgaagCg tCgcCgggtC gcCgtgtgtg taccccCgcc tatgtctgtc tgtcCgCggc CgCgtgtgCg gctgtctgtg gctgtgagcc by direct Sanger sequencing of the PCR amplicons of bisulfite treated DNA. The sequences of the MethPrimers were 5′**-**TTATGAATGTGGTTTTAGAGAG**-**3′ and 5′**-** AACAAAAAAACTCTCTCCAATCTCAC-3′.

### Twelve-lead electrocardiogram measurements

A 12-lead electrocardiogram (ECG) was recorded at a paper speed of 25 mm/s and amplification of 10 mm/mV. We measured the RR, PQ, QRS, and corrected QT (QTc) intervals, and the highest J-point (STJ) and ST segment elevation amplitude in the V1 and V2 leads. The ST morphology in the right precordial leads is known to exhibit day-by-day variations. We therefore recorded ECGs for each participant on at least five different days and the type 1 Brugada ECG was diagnosed when the morphology was recorded spontaneously on at least one occasion [13].

### Signal-averaged ECG findings

Late potentials were analyzed using a FP-705LP system (Fukuda Denshi, Tokyo, Japan). The ECG was recorded during sinus rhythm, using Frank X, Y, and Z leads. The signals from 300 beats were amplified, digitized, averaged, and filtered with backward and forward filters at a high-bandpass frequency of 40 Hz. The filtered QRS (f-QRS) duration, the root mean square voltage of the terminal 40 ms of f-QRS (RMS 40), and the duration of low-amplitude signals <40 μV in the terminal f-QRS (LAS 40) were determined. Late potentials were recognized as being positive when two of the following criteria were satisfied: f-QRS > 114 ms, RMS 40 < 20 μV, or LAS 40 > 38 ms [14].

### Electrophysiological study

Electrophysiological studies were performed in 66 patients using three 5-Fr gage quadripolar electrode catheters with an inter-electrode distance of 5 mm. The catheters were placed at the high right atrium, His bundle, and right ventricular apex. The AH and HV intervals were measured on the baseline ECG. In addition, the sinus node recovery time and atrioventricular node effective refractory period were measured. The patients gave their written informed consent prior to participating in this study.

### Statistical analysis

Data are presented as the mean ± standard deviation. Continuous data were compared between the two groups using unpaired Student’s *t* tests. The χ^2^ test and the Cochran–Armitage trend test were used to assess genetic associations between the cases and controls. Deviation from the Hardy–Weinberg equilibrium was tested among the cases and controls using an ordinary χ^2^ test. A value of *P* < 0.05 was considered statistically significant. The event-free survival curve after birth was constructed following the Kaplan–Meier method, and the results were compared using the log-rank test.

## Results

### Frequency of H558R in BrS cases and controls

**Fig. 1:**
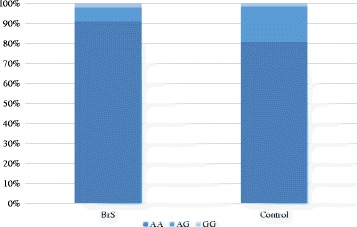
Frequency of H558R in the BrS cases and controls, The minor G allele of *rs1805124* was less frequent in BrS patients than in normal control cases (*P* = 0.028, odds ratio (OR) 1.97)

**Fig. 2:**
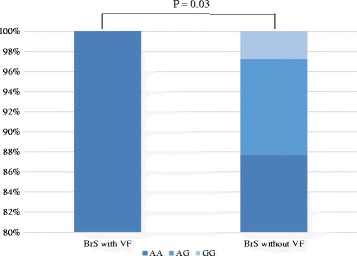
Frequency of H558R in the BrS cases with or without VF history, The G allele (H558R) was not observed in BrS patients with a history of VF (*P* = 0.03)

The frequency of H558R in the BrS cases and controls is shown in Fig. 1. The minor G allele of *rs1805124* was less frequent in BrS patients than in normal control cases (*P* = 0.028, odds ratio (OR): 1.97). Notably, the G allele was not observed in BrS patients with a history of VF (*P* = 0.03; Fig. 2). A comparison of the clinical characteristics in BrS patients with G allele of *rs1805124* (H558R+ group, *N* = 9) or without G allele (H558R- group, *N* = 91) is presented in Table 1. In the H558R+ group, no case had a history of VF or syncope and all of them were asymptomatic.

**Table 1:** Clinical Characteristics in Brugada Syndrome Patients With or Without the G Allele of rs1805124 (H558R+ Group or H558R- Group)

	H558R- group (*N* = 91)	H558R+ group (*N* = 9)	*P* value
Age (years)	44 ± 14	53 ± 12	0.10
Sex (male%)	90.1%	100%	0.20
History of VF	29.7%	0%	0.01
History of syncope	25.3%	0%	0.02
Family history of SCD	35.1%	22.2%	0.43
Atrial Fibrillation	15.4%	33.3%	0.21
Induction of VF	51.6%	77.7%	0.30

*VF* ventricular fibrillation, *SCD* sudden cardiac death

Findings from the 12-lead electrocardiogram, signal-averaged electrocardiogram, and electrophysiological studies are shown in Table 2. Spontaneous type 1 morphology in the V1 was observed less often in the H558R+ group than in the H558R- group (33.3% vs. 74.7%, *P* = 0.01). In addition, the elevation levels of ST segment in the J points of the V1 and V2 leads were significantly lower in the H558R+ group than in the H558R- group (V1: 1.7 ± 0.5 vs. 2.8 ± 1.4, *P* = 0.0003; V2: 2.4 ± 1.3 vs. 4.2 ± 1.8, *P* = 0.0038). There were no significant differences in the other ECG, signal-averaged ECG, and electrophysiological study parameters.

**Table 2:** Twelve-Lead ECG, SAECG, and EPS Findings in Patients With or Without the G Allele of rs1805124 (H558R+ Group or H558R- Group)

	H558R- Group (N = 91)	H558R+ Group (N = 9)	P value
ECG			
Spontaneous type1 Brugada ECG in V1 lead	74.7%	33.3%	0.01
Spontaneous type1 Brugada ECG in V2 lead	40.2%	33.3%	0.68
ST in V1 (mV)	2.8 ± 1.4	1.7 ± 0.5	0.0003
ST in V2 (mV)	4.2 ± 1.8	2.4 ± 1.3	0.0038
J points in V1 (mV)	3.3 ± 2.2	2.3 ± 0.5	0.19
J points in V2 (mV)	4.8 ± 2.4	3.5 ± 1.6	0.12
PQ (ms)	170 ± 33	174 ± 23	0.76
QRS (ms)	120 ± 44	106 ± 16	0.61
QT (ms)	397 ± 33	407 ± 24	0.36
QTc (ms)	413 ± 34	425 ± 23	0.32
SAECG			
Filtered QRS duration (ms)	163 ± 29	155 ± 26	0.48
RMS 40 (μV)	13 ± 10	11 ± 6	0.67
LAS 40 (ms)	50 ± 15	53 ± 14	0.62
Positive late potentials (%)	76.3%	66.6%	0.53
EPS			
AH (ms)	100 ± 20	90 ± 18	0.23
HV (ms)	49 ± 12	48 ± 11	0.77
AVN refractory period (ms)	385 ± 114	306 ± 151	0.12
Sinus node recovery time (ms)	1319 ± 450	1273 ± 87	0.80

*ECG* electrocardiogram, *SAECG* signal-averaged ECG, *EPS* electrophysiological study, *RMS 40* root mean square voltage (40 ms), *LAS 40* low-amplitude signal

### Resequencing results for the *SCN5A* gene in BrS cases

The *SCN5A* mutations were detected in 9 of 100 (9%) BrS patients (H278R, N782 T, N740del, E1784K, G1420R, R1023C, K1859G, A1186T, c.3840 + 1 G > A). They did not coexist with H558R.

### H558R and the first manifestation of VF in BrS cases

**Fig. 3:**
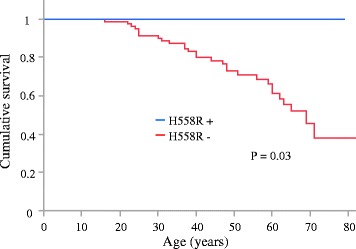
Kaplan–Meier analysis of cumulative survival from VF in 100 BrS patients. The occurrence ratio of VF was significantly lower in the H558R+ group than in the H558R- group according to a log-rank test (*P* = 0.03). VF: ventricular fibrillation; blue line: H558R+ group; red line: H558R- group

VF had occurred in 28 patients prior to enrollment (24 men and 4 women; mean age 40 ± 17 years). After enrollment, during the follow-up period (mean 76 ± 37 months), VF occurred in 9 of the 28 patients with a history of VF and 4 of the 72 patients without VF history. Kaplan–Meier event-free survival curves revealed that the rate of VF events was significantly lower in the BrS patients without history of VF. (Additional file 2: Figure S1, *P* = 0.008 by log rank test) The VF-free survival curves of life-tables method using the Kaplan–Meier demonstrated that BrS patients in the H558R+ group experienced no VF event, and the VF occurrence ratio was significantly lower than for the patients in the H558R- group, according to a log-rank test (*P* = 0.03; Fig. 3).

### Relationship between H558R and the expression and Methylation levels of the *SCN5A* gene

**Fig. 4:**
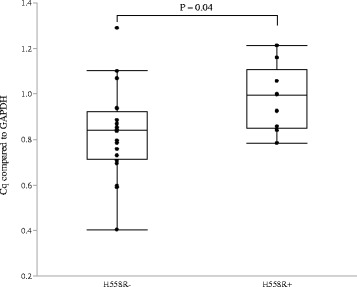
Expression levels of the *SCN5A* gene (using *GAPDH* as a reference gene), according to the presence or absence of H558R. The expression level of *SCN5A* was significantly higher in patients with H558R than in those without

**Fig. 5:**
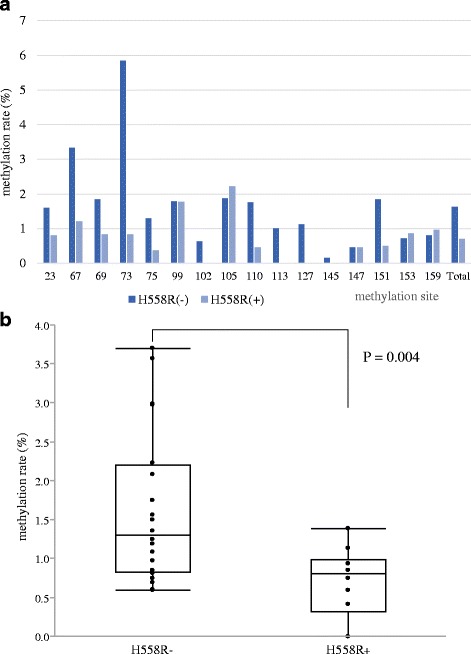
**a** The rate of methylation was lower in 13 of 16 methylation site. The methylation ratio of the *SCN5A* gene, according to the presence or not of H558R. **b** The rate of methylation was lower in patients with H558R than in those without (0.7 ± 0.2% vs. 1.6 ± 0.1%, *P* = 0.004). All data are indicated by box plots and mean ± standard error

**Fig. 6:**
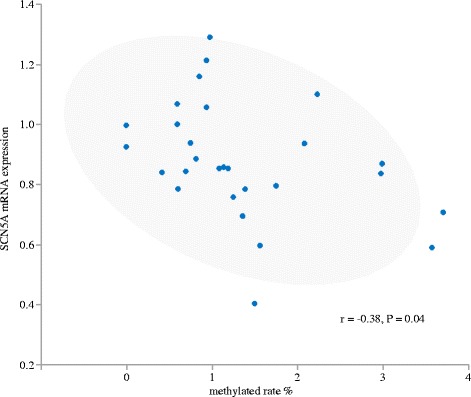
The correlation of mRNA expression and the rate of methylation in SCN5A. There is mild inverse correlation between the mRNA expression and the rate of methylation in SCN5A (r = −0.38, P = 0.04)

The heterozygous H558R was detected in 10 of 30 patients whose right atrial sections were obtained for the expression study. The expression level of *SCN5A* was significant higher in the right atrial sections of patients with H558R (*N* = 10) than of those without (*N* = 20) ((0.98 ± 0.14 vs. 0.83 ± 0.19, *P* = 0.04; Fig. 4). The rate of methylation was lower in 13 of 16 methylation site (Fig. 5a) and the total methylation rate was also lower in patients with H558R than in those without (0.7 ± 0.2% vs. 1.6 ± 0.1%, *P* = 0.004; Fig. 5b). There is mild inverse correlation between the mRNA expression and the rate of methylation in SCN5A (*r* = −0.38, P = 0.04; Fig. 6).

### Expression ratio of G allele /a allele in patients with heterozygous H558R

**Fig. 7:**
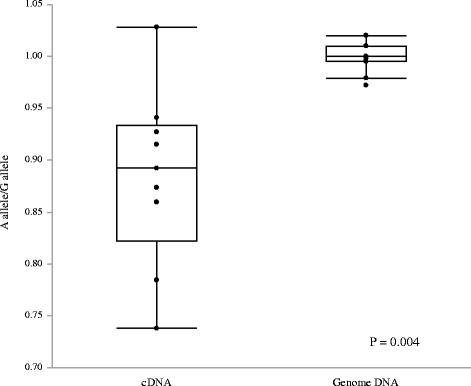
The expression ratio of the A allele and G allele in patients with heterozygous H558R. The expression levels of G allele and A allele was similar in genome DNA but the A allele (wild) expression was 1.38 fold higher than G allele (pathogenic) expression in mRNA. Allelic imbalance existed in mRNA in patients with heterozygous H558R. (*P* = 0.004 vs. genome DNA)

We analyzed expression ratio of the A allele and G allele separately by specific probes in patients with heterozygous H558R.The expression levels of G allele and A allele was similar in genome DNA but the A allele (wild) expression was 1.38 fold higher than G allele (pathogenic) expression in mRNA. Allelic imbalance existed in mRNA in patients with heterozygous H558R. (P = 0.004 vs genome DNA; Fig. 7).

## Discussion

In this study, we compared the frequency of the common *SCN5A* polymorphism H558R (*rs1805124*) in 100 BrS patients and 1875 normal controls. The frequency of H558R was lower in the BrS patients than in the normal controls. Additionally, in the non-*SCN5A* mutation carrier BrS patients with H558R, the existence of H558R improved their ECG findings and they experienced no VF events. Furthermore, *SCN5A* expression was higher and the proportion of *SCN5A* methylation was lower in patients with H558R than in those without. Our results suggest that H558R may provide some protection against the occurrence of VF, even in BrS patients without a *SCN5A* mutation, by modulating *SCN5A* expression and *SCN5A* methylation.

H558R, a known genetic modifier of BrS [3–7], is a common polymorphism of *SCN5A*, with a minor allele frequency of approximately 10% in East Asian populations [15] and nearly 20% in Caucasians. The minor G allele frequency of H558R in the normal controls of the present study was consistent with the previous study just cited. In contrast, the minor G allele frequency was significantly lower in the BrS patients than in the normal controls in both our study and the published control data. There have been a few reports about the frequency of H558R in BrS patients and controls, with one paper reporting no allele difference between BrS patients and normal controls; however, the numbers of cases and controls in that study were small [16]. Another study has reported that the frequency of H558R was lower in a Japanese population with arrhythmia than in normal controls [17]. BrS is known to be more prevalent in East Asians than in Caucasians [13], and the frequency of H558R is lower in East Asians than in Caucasians, as already mentioned. Our result that the frequency of H558R was lower in BrS patients than in normal controls corresponds with this. Additionally, in our study, no BrS patient with H558R suffered a VF event and all were asymptomatic. These findings suggest that the H558R provides some protective effects against the pathogenesis of BrS and the incidence of VF in BrS patients.

According to clinical findings, H558R has been observed to have modulatory effects in BrS patients with pathological mutations [7]. These authors reported that the *SCN5A* mutation carrier of BrS patients with H558R improved their ECG characteristics and was associated with fewer VF events than in patients without H558R. In the present study, we also demonstrated that BrS patients with a H558R polymorphism had lower J-point and ST elevation in the right precordial leads and fewer type 1 ECGs, even in the non-*SCN5A* mutation carriers. Spontaneous type 1 ECG and prominent J-ST elevation are known to be predictors of VF occurrence in BrS [18–20]. Our findings suggested that some modification by H558R of inward or outward channel currents or action potentials had a protective effect on the BrS ECG and on the occurrence of VF.

We therefore went on to investigate the mechanism explaining why H558R had this protective effect on BrS patients. There have been many reports that H558R modifies the loss of the functional effect on *SCN5A* brought by *SCN5A* mutations [3–6]. After Viswanathan et al. reported in 2003 that H558R modified the in vitro effects on the Na^+^ channel function brought by the nearby mutation T512I in the same allele [6], many researchers have reported that H558R improves sodium channel activity in mutated channels by restoring abnormal channel gating kinetics or membrane trafficking [3–5].

Poelzing et al. reported an asymptomatic individual with H558R in a family that carried the R282H-*SCN5A* mutation and showed that coexpression of the mutation with the polymorphism produced a significantly greater current than coexpression of the mutant with the wild-type gene [21]. Many studies have used the patch clamp technique to estimate channel activity derived from *SCN5A* mutation or polymorphisms. Most of these reported that H558R had protective effects on the decreased channel activity that came from *SCN5A*; but whether the H558R itself changed the channel activity, and the mechanisms by which it rescues the *SCN5A* mutations, have proved controversial and as yet have not been fully defined [22, 23].

We performed dd-PCR, more sensitive and quantitative than RT-PCR, using human specimens and demonstrated that the expression level of *SCN5A* mRNA was higher in the participants with H558R than in those without. The increased *SCN5A* expression in people with H558R may be associated with the protective effects of H558R in BrS patients. We also analyzed expression ratio of the A allele and G allele separately by specific probes in patients with heterozygous H558R and found that allelic imbalance existed (pathogenic G allele expression was lower than A allele) in mRNA in patients with heterozygous H558R. We suggested a hypothesis that the mutation effect may prone to be relieved if the heterozygous mutation rode on the risk allele G (Cis allele) (Additional file 3: Figure S2).

For DNA methylation study, we used genomic DNA from cardiac tissue to demonstrate a lower methylation level of *SCN5A* promoters in participants with H558R than in those without. DNA methylation has been reported to be a regulator of specific tissue gene expression [24]. The CpG islands in a promoter lesion are not generally methylated and act as transcription initiation sites [25]. Park et al. reported that *SCN5A* promoter variants were associated with disease severity, but the methylation rate of *SCN5A* using genomic DNA from blood was similar in the patients with or without the *SCN5A* promoter variants [26]. The low methylation rate of CpG islands in SCN5A promoter may contribute to increase the SCN5A expression in patients with H558R than those without.

The linker between DI and DII of SCN5A was reported to be a hot-spot for Arginine methylation. Arginines (R513, R526, and R680) located between DI and DII are reported to be modified by methylation and the mutation of R526H is known to cause Brugada syndrome [27]. In addition, there are phosphorylated serines, including S516, S524 and S525 close to the methylation sites. The R513 methylation and S516 phosphorylation was reported to be reciprocally interacted [28]. The protein arginine methyl transferases (PRMT)-3 and −5 was reported to methylate SCN5A in vitro and increase SCN5A cell surface expression and Na^+^ current density [29]. The R558 also exists between DI and DII and the methylation state may be regulated under the change from H558 to R558 and related to post-translational modification of SCN5A. Tatarinova T et al. reported an inverse relationship between the gene-body and promoter CpG island methylation [30]. The R558 (CGC) may be more prone to being methylated than H558 (CAC) and may be related to a suppressive modification of the SCN5A promoter CpG island methylation.

Given that VF events in BrS patients are rare before reaching adolescence, it is possible that the epigenetics rerates to pathogenesis of VF. The prevalence of several diseases including cancer, type 2 diabetes, metabolic syndrome, cardiovascular disease, and dementia are known to increase with age. Environmental factors are deeply involved in the onset of these disease and the epigenetic changes play an important role in this process [31]. The epigenetic changes in critical genes may contribute to the age-related increase in morbidity of these disease [32]. The VF occurrence in the BrS patients increased after 20 years of age in this study and it is compatible with the hypothesis via methylation difference.

In addition, there is mild inverse correlation between the mRNA expression and the rate of methylation in SCN5A. The precise relationship between H558R and *SCN5A* methylation remains unclear and further investigation will be needed; however, to the best of our knowledge the present study is the first to suggest a protective mechanism of H558R on BrS.

AF incidence rate was similar in the BrS patients with and without H558R in this study. The pathophysiology of AF in BrS has not been completely elucidated. In some reports, SCN5A gain of function mutations have been reported to be linked to an increased susceptibility to AF by enhancing the cellular excitability and lowering the action potential threshold [33, 34].

Kusano K et al. reported that SCN5A mutations were associated with a prolonged intraatrial conduction time, but were not related to the occurrence rate of spontaneous AF [35]. Amin AS et al. reported that the presence of an SCN5A mutation was associated with intra-atrial conduction slowing and suppressed atrial ectopic activity. Intra-atrial conduction slowing may provide a plausible substrate for AF maintenance, while reduced atrial ectopic activity may constitute inhibition of the trigger for AF initiation [36]. The facilitatory and inhibitory effects of SCN5A mutations on AF may be one reason why the AF incidence rate was similar in the BrS with and without SCN5A mutations. Spontaneous AF and VF are closely linked clinically, but their mechanism in BrS patients may not be completely coincidence.

There were several limitations to this study. First, the analysis of gene expression and DNA methylation were performed using atrial tissues from patients who underwent operations for diseases other than BrS, because it was difficult to perform the ventricular biopsy in BrS patients and obtain the amount of tissue required for the analysis. Second, no patient among our participants possessed both H558R and the *SCN5A* mutation. We could not validate previous studies in which H558R mitigates the effect of pathological mutations in *SCN5A*. Conversely, this was the first study to demonstrate that H558R was also protective for BrS patients without *SCN5A* mutations. Further studies are needed to clarify the precise mechanism of the relationship between the H558R polymorphism and gene expression or the DNA methylation of *SCN5A* promoters.

## Conclusion

The H558R polymorphism of the *SCN5A* gene modifies the clinical phenotype of BrS by modulating DNA methylation of *SCN5A* gene promoters.

## Additional files

Additional file 1: Table S1. SCN5A primer list. (XLSX 11 kb)

Additional file 2: Figure S1. Kaplan–Meier event-free survival curves revealed that the rate of VF events was significantly lower in the BrS patients without history of VF. (*P* = 0.008 by log rank test). (PPTX 43 kb)

Additional file 3: Figure S2. We suggested a hypothesis that the mutation effect may prone to be relieved if the heterozygous mutation rode on the risk allele G (Cis allele) but actualized if they exist on trans allele. (PPTX 39 kb)

## Abbreviations

AH: Right atria to His
BrS: Brugada syndrome
dd-PCR: Droplet Digital polymerase chain reaction
ECG: Electrocardiogram
f-QRS: Filtered QRS
GAPDH: Glyceraldehyde-3-phosphate dehydrogenase
HV: His to right ventricule
LAS 40: Low-amplitude signals <40 μV in the terminal f-QRS
OR: Odds ratio
PCR: Polymerase chain reaction
RMS 40: Root mean square voltage of the terminal 40 ms of f-QRS
RT-PCR: Reverse transcription polymerase chain reaction
SCN5A: Sodium voltage-gated channel alpha subunit 5
SNP: Single nucleotide polymorphism
VF: Ventricular fibrillation

## References

[1] Brugada P, Brugada J (1992). Right bundle branch block, persistent ST segment elevation and sudden cardiac death: a distinct clinical and electrocardiographic syndrome: a multicenter report. J Am Coll Cardiol.

[2] Chen Q, Kirsch GE, Zhang D, Brugada R, Brugada J, Brugada P, Potenza D, Moya A, Borggrefe M, Breithardt G, Ortiz-Lopez R (1998). Genetic basis and molecular mechanism for idiopathic ventricular fibrillation. Nature.

[3] Shinlapawittayatorn K, Dudash LA, Du XX, Heller L, Poelzing S, Ficker E, Deschênes I (2011). A novel strategy using cardiac sodium channel polymorphic fragments to rescue trafficking-deficient SCN5A mutations. Circ Cardiovasc Genet.

[4] Shinlapawittayatorn K, Du XX, Liu H, Ficker E, Kaufman ES, Deschênes I (2011). A common SCN5A polymorphism modulates the biophysical defects of SCN5A mutations. Heart Rhythm.

[5] Ye B, Valdivia CR, Ackerman MJ, Makielski JC (2003). A common human SCN5A polymorphism modifies expression of an arrhythmia causing mutation. Physiol Genomics.

[6] Viswanathan PC, Benson DW, Balser JR (2003). A common SCN5A polymorphism modulates the biophysical effects of an SCN5A mutation. J Clin Invest.

[7] Lizotte E, Junttila MJ, Dube MP, Hong K, Benito B, De Zutter M, Henkens S, Sarkozy A, Huikuri HV, Towbin J, Vatta M (2009). Genetic modulation of brugada syndrome by a common polymorphism. J Cardiovasc Electrophysiol.

[8] Priori SG, Wilde AA, Horie M, Cho Y, Behr ER, Berul C, Blom N, Brugada J, Chiang CE, Huikuri H, Kannankeril P (2014). HRS/EHRA/APHRS expert consensus statement on the diagnosis and management of patients with inherited primary arrhythmia syndromes. J Arrhythm.

[9] Ohnishi Y, Tanaka T, Ozaki K, Yamada R, Suzuki H, Nakamura Y (2001). A high-throughput SNP typing system for genome-wide association studies. J Hum Genet.

[10] Suzuki A, Yamada R, Chang X, Tokuhiro S, Sawada T, Suzuki M, Nagasaki M, Nakayama-Hamada M, Kawaida R, Ono M, Ohtsuki M (2003). Functional haplotypes of PADI4, encoding citrullinating enzyme peptidylarginine deiminase 4, are associated with rheumatoid arthritis. Nat Genet.

[11] Nakano Y, Ochi H, Onohara Y, Toshishige M, Tokuyama T, Matsumura H, Kawazoe H, Tomomori S, Sairaku A, Watanabe Y, Ikenaga H (2016). Common variant near HEY2 has a protective effect on ventricular fibrillation occurrence in Brugada syndrome by regulating the repolarization current. Circ Arrhythm Electrophysiol.

[12] Djurisic S, Teiblum S, Tolstrup CK, Christiansen OB, Hviid TV (2015). Allelic imbalance modulates surface expression of the tolerance-inducing HLA-G molecule on primary trophoblast cells. Mol Hum Reprod.

[13] Antzelevitch C (2005). Heart Rhythm Society; European heart rhythm association. Brugada syndrome: report of the second consensus conference: endorsed by the Heart Rhythm Society and the European heart rhythm association. Circulation.

[14] Breithardt G, Cain ME, El-Sherif N, Flowers NC, Hombach V, Janse M, Simson MB, Steinbeck G (1991). Standards for analysis of ventricular late potentials using high-resolution or signal-averaged electrocardiography: a statement by a task force committee of the European Society of Cardiology, the American Heart Association, and the American College of Cardiology. J Am Coll Cardiol.

[15] Ackerman MJ, Splawski I, Makielski JC, Tester DJ, Will ML, Timothy KW, Keating MT, Jones G, Chadha M, Burrow CR, Stephens JC (2004). Spectrum and prevalence of cardiac sodium channel variants among black, white, Asian, and Hispanic individuals: implications for arrhythmogenic susceptibility and Brugada/long QT syndrome genetic testing. Heart Rhythm.

[16] Chen JZ, Xie XD, Wang XX, Tao M, Shang YP, Guo XG (2004). Single nucleotide polymorphisms of the SCN5A gene in Han Chinese and their relation with Brugada syndrome. Chin Med J.

[17] Maekawa K, Saito Y, Ozawa S, Adachi-Akahane S, Kawamoto M, Komamura K, Shimizu W, Ueno K, Kamakura S, Kamatani N, Kitakaze M (2005). Genetic polymorphisms and haplotypes of the human cardiac sodium channel α subunit gene (SCN5A) in Japanese and their association with arrhythmia. Ann Hum Genet.

[18] Brugada J, Brugada R, Antzelevitch C, Towbin J, Nademanee K, Brugada P (2002). Long-term follow-up of individuals with the electrocardiographic pattern of right bundle-branch block and ST-segment elevation in precordial leads V1 to V3. Circulation.

[19] Priori SG, Napolitano C, Gasparini M, Pappone C, Della Bella P, Giordano U, Bloise R, Giustetto C, De Nardis R, Grillo M, Ronchetti E (2002). Natural history of Brugada syndrome insights for risk stratification and management. Circulation.

[20] Fish JM, Antzelevitch C (2004). Role of sodium and calcium channel block in unmasking the Brugada syndrome. Heart Rhythm.

[21] Poelzing S, Forleo C, Samodell M, Dudash L, Sorrentino S, Anaclerio M, Troccoli R, Iacoviello M, Romito R, Guida P, Chahine M (2006). SCN5A polymorphism restores trafficking of a Brugada syndrome mutation on a separate gene. Circulation.

[22] Cheng J, Morales A, Siegfried JD, Li D, Norton N, Song J, Gonzalez-Quintana J, Makielski JC, Hershberger RE (2010). SCN5A rare variants in familial dilated cardiomyopathy decrease peak sodium current depending on the common polymorphism H558R and common splice variant Q1077del. Clin Transl Sci.

[23] Tan BH, Valdivia CR, Rok BA, Ye B, Ruwaldt KM, Tester DJ, Ackerman MJ, Makielski JC (2005). Common human SCN5A polymorphisms have altered electrophysiology when expressed in Q1077 splice variants. Heart Rhythm.

[24] Jones PA, Takai D (2001). The role of DNA methylation in mammalian epigenetics. Science.

[25] Li E, Beard C, Jaenisch R (1993). Role for DNA methylation in genomic imprinting. Nature.

[26] Park JK, Martin LJ, Zhang X, Jegga AG, Benson DW (2012). Genetic variants in SCN5A promoter are associated with arrhythmia phenotype severity in patients with heterozygous loss-of-function mutation. Heart Rhythm.

[27] Beltran-Alvarez P, Pagans S, Brugada R (2011). The cardiac sodium channel is post-translationally modified by arginine methylation. J Proteome Res.

[28] Marionneau C, Abriel H (2015). Regulation of the cardiac Na+ channel NaV1.5 by post-translational modifications. J Mol Cell Cardiol.

[29] Beltran-Alvarez P, Espejo A, Schmauder R, Beltran C, Mrowka R, Linke T, Batlle M, Pérez-Villa F, Pérez GJ, Scornik FS, Benndorf K (2013). Protein arginine methyl transferases-3 and -5 increase cell surface expression of cardiac sodium channel. FEBS Lett.

[30] Tatarinova T, Elhaik E, Pellegrini M (2013). Cross-species analysis of genic GC3 content and DNA methylation patterns. Genome Biol Evol.

[31] Heerwagen MJ, Miller MR, Barbour LA, Friedman JE (2010). Maternal obesity and fetal metabolic programming: a fertile epigenetic soil. Am J Physiol Regul Integr Comp Physiol.

[32] Ali O, Cerjak D, Kent JW, James R, Blangero J, Carless MA, Zhang Y (2015). An epigenetic map of age-associated autosomal loci in northern European families at high risk for the metabolic syndrome. Clin Epigenetics.

[33] Savio-Galimberti E, Darbar D (2014). Atrial fibrillation and SCN5A variants. Card Electrophysiol Clin.

[34] Li Q, Huang H, Liu G, Lam K, Rutberg J, Green MS, Birnie DH, Lemery R, Chahine M, Gollob MH (2009). Gain-of-function mutation of Nav1.5 in atrial fibrillation enhances cellular excitability and lowers the threshold for action potential firing. Biochem Biophys Res Commun.

[35] Kusano KF, Taniyama M, Nakamura K, Miura D, Banba K, Nagase S, Morita H, Nishii N, Watanabe A, Tada T, Murakami M (2008). Atrial fibrillation in patients with Brugada syndrome relationships of gene mutation, electrophysiology, and clinical backgrounds. J Am Coll Cardiol.

[36] Amin AS, Boink GJ, Atrafi F, Spanjaart AM, Asghari-Roodsari A, Molenaar RJ, Ruijter JM, Wilde AA, Tan HL (2011). Facilitatory and inhibitory effects of SCN5A mutations on atrial fibrillation in Brugada syndrome. Europace.

