# Supplementary material for: H558R, a common SCN5A polymorphism, modifies the clinical phenotype of Brugada syndrome by modulating DNA methylation of SCN5A promoters
Source: J Biomed Sci. 2017 Dec 4;24:91. doi: 10.1186/s12929-017-0397-x (PMC5713129; doi:10.1186/s12929-017-0397-x)
Supplement: Supplementary file 2 — Kaplan–Meier event-free survival curves revealed that the rate of VF events was significantly lower in the BrS patients without history of VF. (P = 0.008 by log rank test). (PPTX 43 kb) [file 12929_2017_397_MOESM2_ESM.pptx]

## Slide 1
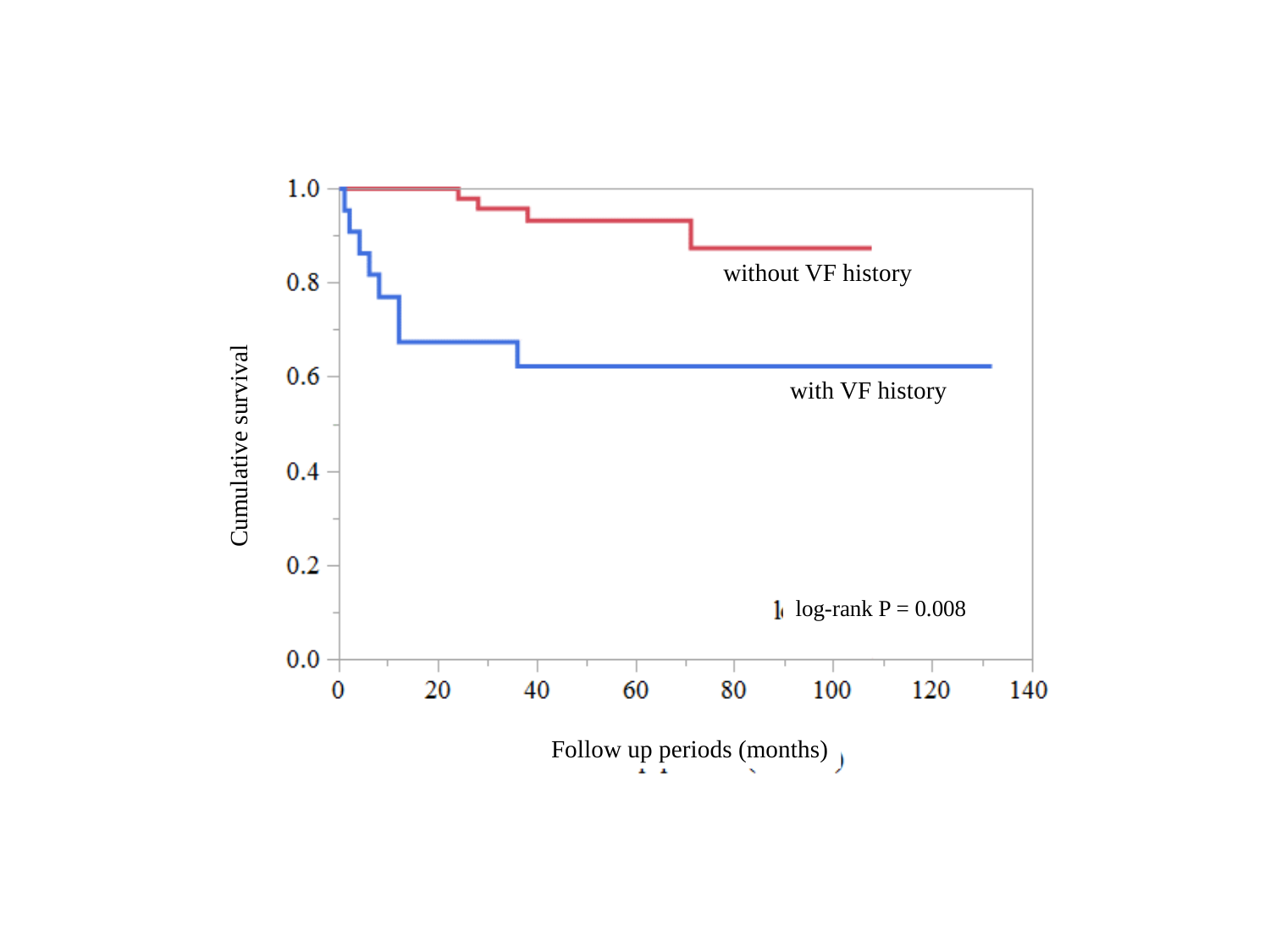

without VF history
with VF history
Cumulative survival
log-rank P = 0.008
Follow up periods (months)
