# Supplementary material for: H558R, a common SCN5A polymorphism, modifies the clinical phenotype of Brugada syndrome by modulating DNA methylation of SCN5A promoters
Source: J Biomed Sci. 2017 Dec 4;24:91. doi: 10.1186/s12929-017-0397-x (PMC5713129; doi:10.1186/s12929-017-0397-x)
Supplement: Supplementary file 3 — We suggested a hypothesis that the mutation effect may prone to be relieved if the heterozygous mutation rode on the risk allele G (Cis allele) but actualized if they exist on trans allele. (PPTX 39 kb) [file 12929_2017_397_MOESM3_ESM.pptx]

## Slide 1
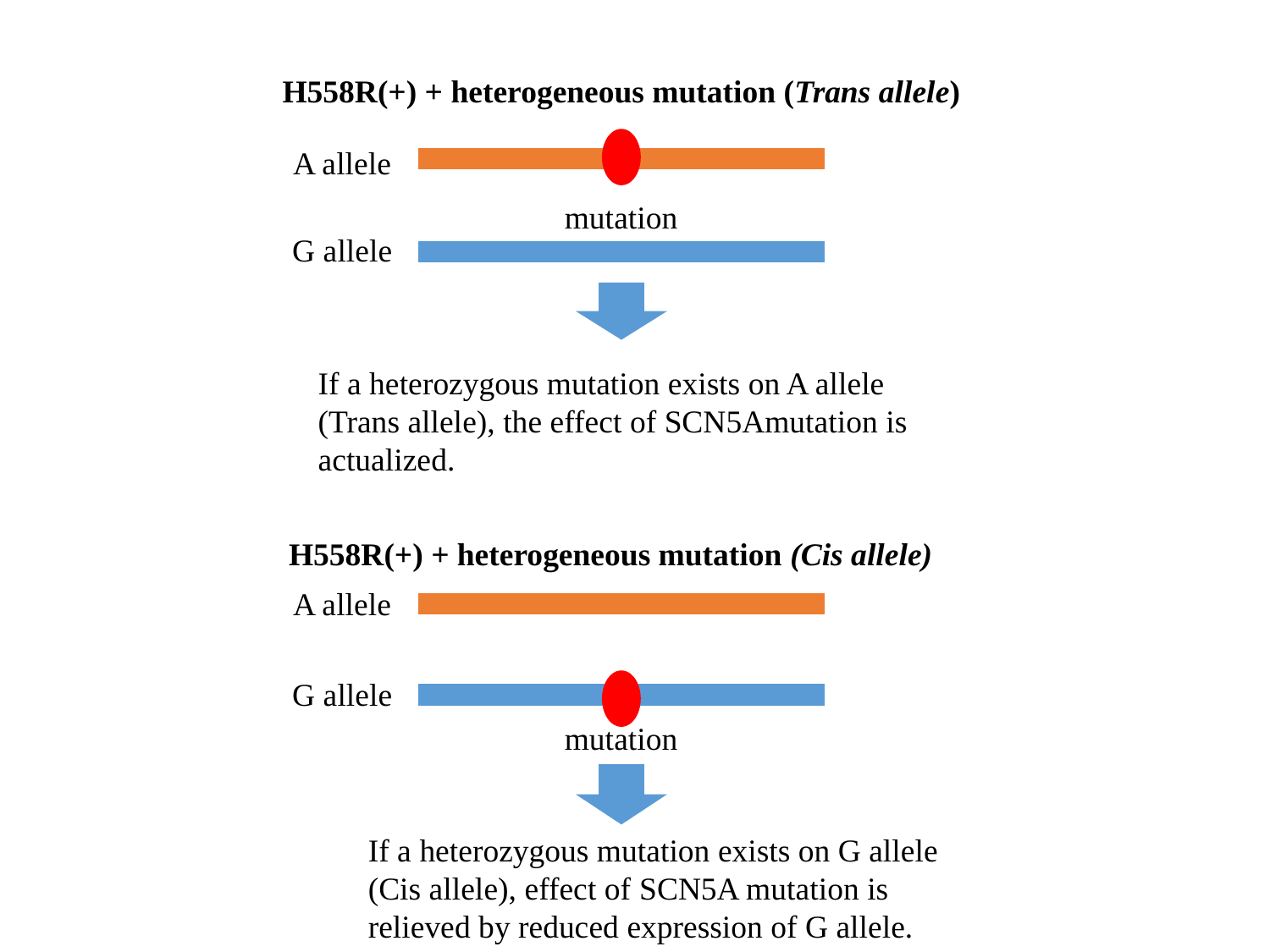

H558R(+) + heterogeneous mutation (Trans allele)
A allele
mutation
G allele
If a heterozygous mutation exists on A allele (Trans allele), the effect of SCN5Amutation is actualized.
H558R(+) + heterogeneous mutation (Cis allele)
A allele
G allele
mutation
If a heterozygous mutation exists on G allele (Cis allele), effect of SCN5A mutation is relieved by reduced expression of G allele.
